# Study of Vitamin D and Serum Calcium Levels in Type 2 Diabetes Mellitus

**DOI:** 10.7759/cureus.108040

**Published:** 2026-04-30

**Authors:** Ninad Shinde, Vijaykumar G Warad, Shravankumar Potkar, Shridhar Patil

**Affiliations:** 1 General Medicine, Shri B.M. Patil Medical College Hospital and Research Centre, Bijapur Lingayat District Educational Association (BLDE) (Deemed to be University), Vijayapura, IND

**Keywords:** glycaemic control, hba1c, serum calcium, type 2 diabetes mellitus, vitamin d

## Abstract

Introduction: Type 2 diabetes mellitus (T2DM) is a growing global health concern. Emerging evidence suggests that vitamin D and calcium play an important role in glucose metabolism and insulin function. The present study aimed to compare serum vitamin D and calcium levels between patients with T2DM and healthy controls and evaluate their association with glycaemic control (HbA1c).

Materials and methods: This hospital-based case-control study included 136 participants, comprising 68 patients with T2DM and 68 healthy controls. Serum vitamin D and calcium levels were estimated, along with fasting blood sugar (FBS), postprandial blood sugar (PPBS), and glycated haemoglobin (HbA1c). Vitamin D and calcium status were categorized using standard cut-offs. Statistical analysis was performed using an independent t-test, chi-square test, and Pearson correlation.

Results: Glycaemic parameters were significantly higher in diabetic patients, including FBS (149.7 ± 51.0 vs. 87.2 ± 14.2 mg/dL), PPBS (223.5 ± 77.3 vs. 123.5 ± 17.6 mg/dL), and HbA1c (9.4 ± 2.1% vs. 5.1 ± 0.4%) (p = 0.001). Serum calcium (8.3 ± 0.7 vs. 9.0 ± 0.6 mg/dL) and vitamin D (20.23 ± 9.07 vs. 25.21 ± 8.23 ng/mL) were significantly lower in diabetics (p = 0.001). Vitamin D deficiency was more prevalent in diabetic patients (34 (50.0%) vs. 20 (29.4%); p = 0.038), and hypocalcaemia was also significantly higher (38 (55.9%) vs. 18 (26.5%); p = 0.001). Serum vitamin D (r = −0.412, p = 0.001) and calcium (r = −0.356, p = 0.002) showed significant negative correlation with HbA1c.

Conclusion: Patients with T2DM have significantly lower serum vitamin D and calcium levels, which are associated with poor glycaemic control. Assessment of vitamin D and calcium status may help identify micronutrient abnormalities in patients with T2DM; however, interventional studies are required to determine clinical benefit.

## Introduction

Diabetes mellitus is a major global health concern, with a rapidly increasing prevalence worldwide [1]. It is estimated that over 150 million individuals are currently affected, and this number is projected to rise to nearly 439 million by 2030, with type 2 diabetes mellitus (T2DM) accounting for the majority of cases [2,3]. In India, the burden is particularly high, with approximately 77 million individuals affected in 2019 and projections suggesting a rise to 134 million by 2045 [4]. This escalating prevalence poses a significant challenge to healthcare systems due to its associated morbidity and long-term complications [5].

T2DM is a chronic metabolic disorder characterized by persistent hyperglycaemia resulting from impaired insulin secretion, insulin resistance, or both [6,7]. While traditional risk factors such as obesity, sedentary lifestyle, and genetic predisposition are well established, growing evidence suggests that micronutrients, particularly vitamin D and calcium, play an important role in glucose homeostasis [8,9]. These factors have been increasingly recognized for their potential influence on both insulin secretion and peripheral insulin sensitivity [10].

Vitamin D exerts its effects on glucose metabolism by enhancing insulin sensitivity through upregulation of insulin receptor expression and facilitation of glucose transport into cells [11]. Additionally, vitamin D plays a crucial role in maintaining calcium homeostasis, which is essential for insulin-mediated intracellular processes [12]. Calcium is involved in insulin secretion from pancreatic β-cells and is necessary for proper insulin signalling [13]. Alterations in calcium flux and impaired intracellular calcium regulation may lead to defective insulin action and contribute to insulin resistance [14].

Recent studies have demonstrated an association between low vitamin D levels, altered calcium metabolism, and poor glycaemic control [15,16]. However, data remain variable, particularly in the Indian population. Furthermore, the combined relationship of vitamin D and calcium with glycaemic control remains incompletely characterized in this setting. In this context, the present study aimed to (1) compare serum vitamin D and calcium levels between patients with T2DM and healthy controls, and (2) evaluate their association with glycaemic control (HbA1c).

This research work was originally conducted as part of a postgraduate dissertation submitted to the Department of General Medicine, BLDE (Deemed to be University), Vijayapura, Karnataka, India.

## Materials and methods

This hospital-based case-control study was conducted at Bijapur Lingayat District Educational Association (BLDE) (Deemed to be University) Shri B. M. Patil Medical College Hospital and Research Centre over a period of 18 months from March 2024 to September 2025. Ethical approval was obtained from the Institutional Ethics Committee of BLDE (IEC No: BLDE (DU)/IEC-SBMPMC/0465/2023-24). The study was carried out in the Department of General Medicine, including both outpatient and inpatient participants. Written informed consent was obtained from all subjects prior to enrolment.

A total of 136 participants were included and divided into two groups: 68 patients with T2DM (cases) and 68 apparently healthy individuals (controls). Diagnosis of T2DM was established according to the criteria of the American Diabetes Association; the most recent Standards of Care were used for reference [17]. Inclusion criteria for cases comprised patients with confirmed T2DM, while controls were individuals without diabetes and with normal glycaemic parameters. Exclusion criteria included patients with chronic kidney disease, those receiving diuretics or calcium channel blockers, individuals on vitamin D or calcium supplementation, and pregnant women. Patients with chronic kidney disease were excluded based on clinical history and available medical records; renal function parameters were not systematically compared between groups.

Sample size was calculated using G*Power software version 3.1.9.4 (Heinrich Heine University Düsseldorf, Düsseldorf, Germany) based on the study by Bayani MA, which reported mean serum vitamin D levels of 18.7 ± 10.2 ng/mL in cases and 24.6 ± 13.5 ng/mL in controls [18]. The calculation was performed using the formula for the comparison of two independent means: $n = \frac{2 (Z_{\alpha/2} + Z_{\beta})^2 \sigma^2}{d^2}$, where σ represents the pooled standard deviation and d represents the difference between group means. A total sample size of 136 (68 per group) was required to achieve 80% power at a 5% level of significance (α = 0.05).

Data were collected using a predesigned pro forma that included demographic details and clinical history. Pallor was assessed clinically based on conjunctival examination by the treating physician. Venous blood samples (5 mL) were collected under aseptic precautions. Fasting blood sugar (FBS) and postprandial blood sugar (PPBS) were measured using standard enzymatic methods, and glycated haemoglobin (HbA1c) was estimated by high-performance liquid chromatography (HPLC).

Serum calcium was measured using a colorimetric method with a dry chemistry analyzer and graded as low (<8.5 mg/dL), normal (8.5-10.5 mg/dL), and high (>10.5 mg/dL) [19]. Serum 25-hydroxyvitamin D levels were estimated using the chemiluminescent immunoassay (CLIA) technique and categorized as deficient (<20 ng/mL), insufficient (20-29 ng/mL), and sufficient (≥30 ng/mL) [20].

Statistical analysis was performed using IBM SPSS Statistics for Windows, Version 26 (Released 2019; IBM Corp., Armonk, New York). Continuous variables were expressed as mean ± standard deviation, while categorical variables were presented as N (%). Comparisons between groups were performed using the independent samples t-test and chi-square test. The Pearson correlation coefficient was used to assess relationships between continuous variables. A p-value of <0.05 was considered statistically significant.

## Results

The mean age was comparable between the diabetes mellitus and control groups (58.3 ± 11.7 vs. 60.8 ± 15.5 years; p = 0.296). Gender distribution was similar (male: 39 (57.4%) vs. 43 (63.2%); female: 29 (42.6%) vs. 25 (36.8%)) (p = 0.490). Hypertension was significantly higher in diabetic patients (36 (52.9%) vs. 11 (16.2%); p = 0.001), while pallor showed no significant difference (2 (2.9%) vs. 0 (0.0%); p = 0.154) (Table 1).

**Table 1:** Baseline Characteristics and Clinical Profile Data are presented as mean ± standard deviation (SD) for continuous variables and N (%) for categorical variables. Comparison between groups was performed using the independent Student’s t-test for continuous variables and the chi-square (χ²) test for categorical variables. p < 0.05 was considered statistically significant. Abbreviations: SD – Standard Deviation; χ² – Chi-Square Test.

Variable		Diabetes Mellitus (n = 68)	Healthy Controls (n = 68)	Test Value	p-value
Age (years)	Mean ± SD	58.3 ± 11.7	60.8 ± 15.5	t = 1.06	0.296
Gender	Male	39 (57.4%)	43 (63.2%)	χ² = 0.47	0.490
	Female	29 (42.6%)	25 (36.8%)		
Hypertension	Present	36 (52.9%)	11 (16.2%)	χ² = 19.80	0.001
Pallor	Present	2 (2.9%)	0 (0.0%)	χ² = 2.03	0.154

Glycaemic parameters were significantly elevated in the diabetes group, including FBS (149.7 ± 51.0 vs. 87.2 ± 14.2 mg/dL), PPBS (223.5 ± 77.3 vs. 123.5 ± 17.6 mg/dL), and HbA1c (9.4 ± 2.1% vs. 5.1 ± 0.4%) (p = 0.001 for all). Serum calcium (8.3 ± 0.7 vs. 9.0 ± 0.6 mg/dL) and vitamin D (20.23 ± 9.07 vs. 25.21 ± 8.23 ng/mL) were significantly lower in diabetic patients (p = 0.001) (Table 2).

**Table 2:** Comparison of Biochemical Parameters Between Groups Data are expressed as mean ± SD. Intergroup comparisons were performed using the independent Student’s t-test. A p-value < 0.05 was considered statistically significant. Abbreviations: FBS – Fasting Blood Sugar; PPBS – Postprandial Blood Sugar; HbA1c – Glycated Haemoglobin; SD – Standard Deviation.

Variable	Diabetes Mellitus (n = 68)	Healthy Controls (n = 68)	t-value	p-value
FBS (mg/dL)	149.7 ± 51.0	87.2 ± 14.2	7.41	0.001
PPBS (mg/dL)	223.5 ± 77.3	123.5 ± 17.6	8.25	0.001
HbA1c (%)	9.4 ± 2.1	5.1 ± 0.4	12.64	0.001
Serum Calcium (mg/dL)	8.3 ± 0.7	9.0 ± 0.6	6.30	0.001
Serum Vitamin D (ng/mL)	20.23 ± 9.07	25.21 ± 8.23	3.35	0.001

Vitamin D deficiency was more common in the diabetes group (34 (50.0%) vs. 20 (29.4%)), while sufficient levels were higher in controls (20 (29.4%) vs. 12 (17.6%)). The difference in distribution was statistically significant (p = 0.038) (Table 3).

**Table 3:** Vitamin D Status Distribution Data are presented as N (%). Group comparisons were performed using the chi-square (χ²) test. A p-value < 0.05 was considered statistically significant. Vitamin D status was categorized as deficient (<20 ng/mL), insufficient (20–29 ng/mL), and sufficient (≥30 ng/mL) [20]. Abbreviations: χ² – Chi-square test.

Vitamin D Status	Diabetes Mellitus (n = 68)	Healthy Controls (n = 68)	χ²-value	p-value
Deficient (<20 ng/mL)	34 (50.0%)	20 (29.4%)	6.52	0.038
Insufficient (20-29 ng/mL)	22 (32.4%)	28 (41.2%)		
Sufficient (≥30 ng/mL)	12 (17.6%)	20 (29.4%)		

Low serum calcium was observed in 38 (55.9%) diabetic patients compared to 18 (26.5%) controls, whereas normal levels were higher in controls (50 (73.5%) vs. 30 (44.1%)). This difference was statistically significant (p = 0.001) (Table 4).

**Table 4:** Calcium Status Distribution Data are presented as N (%). Comparison between groups was performed using the chi-square (χ²) test. A p-value < 0.05 was considered statistically significant. Serum calcium was categorized as low (<8.5 mg/dL) and normal (≥8.5 mg/dL) [19]. Abbreviations: χ² – Chi-Square Test.

Calcium Status	Diabetes Mellitus (n = 68)	Healthy Controls (n = 68)	χ²-value	p-value
Low (<8.5 mg/dL)	38 (55.9%)	18 (26.5%)	11.84	0.001
Normal (≥8.5 mg/dL)	30 (44.1%)	50 (73.5%)		

Serum vitamin D (r = -0.412, p = 0.001) and calcium (r = -0.356, p = 0.002) showed significant negative correlation with HbA1c, while vitamin D positively correlated with calcium (r = 0.305, p = 0.001) (Table 5).

**Table 5:** Correlation of Serum Vitamin D, Serum Calcium, and HbA1c Correlation analysis was performed using Pearson’s correlation coefficient (r). A p-value < 0.05 was considered statistically significant. Abbreviations: HbA1c – Glycated Haemoglobin; r – Correlation Coefficient.

Variables	Correlation Coefficient (r)	p-value
Serum Vitamin D vs. HbA1c	-0.412	0.001
Serum Calcium vs. HbA1c	-0.356	0.002
Serum Vitamin D vs. Serum Calcium	0.305	0.001

## Discussion

The present study demonstrated comparable baseline characteristics between the diabetes mellitus and control groups in terms of age and gender distribution, indicating appropriate group matching. However, hypertension was significantly more prevalent among diabetic patients (36 (52.9%) vs. 11 (16.2%); p = 0.001), reflecting the known association between diabetes and cardiovascular risk factors. Differences in comorbidities, such as hypertension, between groups may have acted as confounding factors and were not adjusted for in the analysis. Similar demographic patterns have been reported by Jamali et al. and Sarwar et al., where most participants were middle-aged, with no significant differences in baseline characteristics between groups [21,22]. These findings support the validity of the present study population for comparative analysis.

In the current study, glycaemic parameters, including fasting blood sugar, PPBS, and HbA1c, were significantly elevated in diabetic patients compared to controls (p = 0.001), indicating poor glycaemic control. These findings are consistent with studies by Nigah et al. and Chaudhary et al., which also reported significantly higher glucose indices among individuals with T2DM [23,24]. For instance, Chaudhary et al. observed markedly elevated FPG and PPPG levels in diabetic subjects compared to controls (p < 0.001), reinforcing the metabolic dysregulation associated with diabetes [24]. Furthermore, Mahmood et al. demonstrated a negative correlation between vitamin D levels and glycaemic markers, supporting an association between micronutrient status and glucose metabolism [25].

A key finding of the present study was the significantly lower serum vitamin D and calcium levels among diabetic patients compared to healthy controls (p = 0.001). Vitamin D deficiency was observed in 34 (50.0%) diabetic patients, indicating a substantial burden of hypovitaminosis D. These findings are in agreement with studies by Jamali AA and Kumar PS, which reported a high prevalence of vitamin D deficiency among individuals with T2DM [21,26]. Jamali et al. further demonstrated a significant inverse correlation between vitamin D levels and HbA1c (r = −0.314; p = 0.016), indicating an association between lower vitamin D levels and poorer glycaemic control [21]. Similarly, Sarwar et al. reported that both vitamin D and calcium deficiencies were significantly associated with higher HbA1c levels, suggesting a relationship with glycaemic regulation [22].

In addition, the present study identified a significant positive correlation between serum vitamin D and calcium levels (r = 0.305; p = 0.001), along with a moderate negative correlation of both parameters with HbA1c. These findings are comparable to those reported by Chaudhary et al., who observed a positive association between vitamin D and calcium (r = 0.344; p < 0.001), and by Sarwar et al., who demonstrated an inverse relationship of these micronutrients with glycaemic indices [22,24]. Collectively, these observations suggest an association between lower vitamin D and calcium levels and poorer glycaemic control in patients with T2DM. However, due to the observational nature of the study, a causal relationship cannot be established.

Strengths and limitations

The study is strengthened by its well-designed case-control methodology with equal group distribution, use of standardized laboratory methods, and comprehensive analysis including both comparisons and correlations of vitamin D, calcium, and glycaemic parameters. However, its single-centre design and relatively small sample size may limit generalizability. Additionally, the cross-sectional nature precludes causal inference. Important confounders such as body mass index, dietary intake, sunlight exposure, and physical activity were not assessed. Serum calcium was not corrected for albumin levels, which may have influenced the accuracy of hypocalcaemia assessment. Furthermore, differences in comorbid conditions, such as hypertension, between groups were not adjusted for and may have affected the observed associations.

## Conclusions

The present study demonstrates that patients with T2DM have significantly lower serum vitamin D and calcium levels compared to healthy individuals, along with a higher prevalence of vitamin D deficiency and hypocalcaemia. Both vitamin D and calcium showed a significant inverse relationship with HbA1c, indicating their association with poor glycaemic control. Additionally, a positive correlation between vitamin D and calcium highlights their interdependent role in metabolic regulation. These findings suggest that assessment of vitamin D and calcium status may help identify common micronutrient abnormalities in patients with T2DM; however, interventional studies are required to determine clinical benefit.
